# Benign Migratory Glossitis in a Patient With Cholangiocarcinoma due to Targeted Drug Therapy

**DOI:** 10.1002/ccr3.72813

**Published:** 2026-05-25

**Authors:** Sandeep Gurav, Gurkaran Preet Singh, Radhika Jain

**Affiliations:** ^1^ Dental and Prosthetic Surgery, Tata Memorial Hospital A CI of Homi Bhabha National Institute Mumbai India

**Keywords:** angiogenesis inhibitors, benign migratory, bevacizumab, cholangiocarcinoma, drug effects, glossitis

## Abstract

During the course of targeted drug therapy, a cancer patient may manifest a myriad of systemic and oral toxicities. This has a direct impact on the patient's QOL. This clinical image highlights a rarely‐encountered oral mucosal alteration called benign migratory glossitis in a case of cholangiocarcinoma who was subjected to bevacizumab.

## Graphical abstract

Oral Manifestation of Targeted Therapy: Benign Migratory Glossitis.

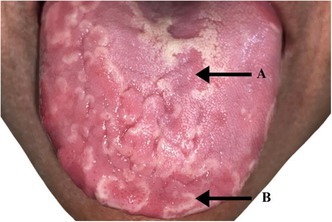

## 1 Introduction

Targeted drug therapy aims to destroy cancer‐causing genetic mutations or proteins that cause uncontrolled cell growth. In spite of being precise in its action, multiple oral and cutaneous toxicities are observed during the course [1]. This has a direct impact on patient compliance and adherence to established protocols, thereby compromising drug delivery and disease resolution.

## 2 Case Presentation

**FIGURE 1:**
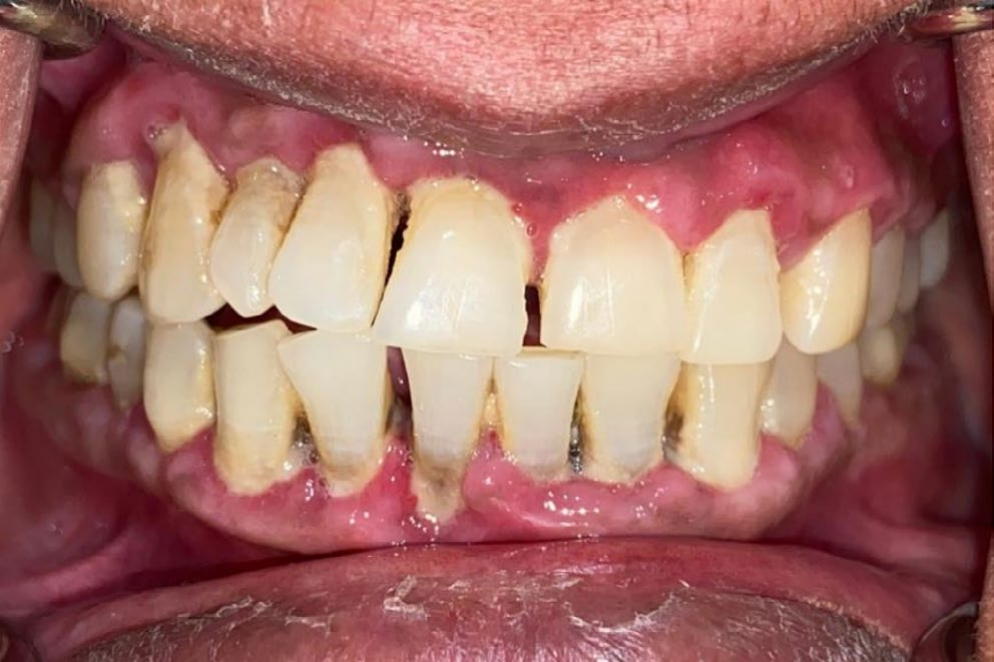
The figure demonstrates the oral hygiene status with periodontally compromised mandibular anterior teeth.

**FIGURE 2:**
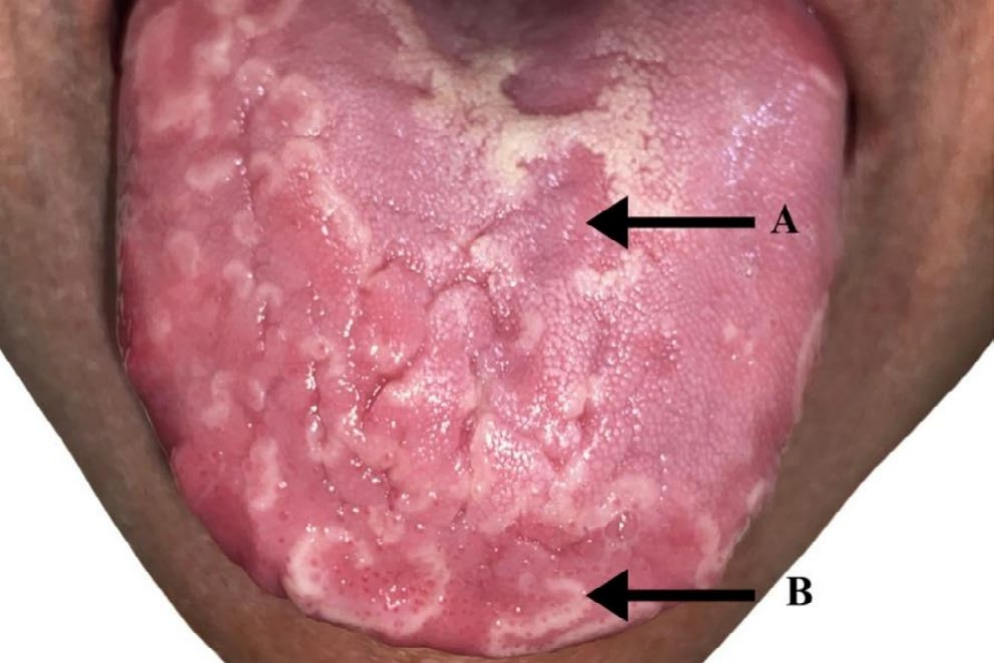
The image depicts the red, desquamated areas (A) surrounded by white regenerating filiform papilla (B). The lesions are spread all over the dorsum and lateral borders of the tongue.

A 49‐year‐old Indian female reported to the dental clinic with a chief complaint of bleeding gums, occasional gum purulence, and sensitivity to hot and spicy foods. She had been undergoing treatment for intrahepatic cholangiocarcinoma. She was subjected to targeted therapy (switch maintenance) comprising of 3‐weekly bevacizumab and a daily dose of erlotinib [2]. Figure 1 demonstrates oral hygiene status with periodontally compromised mandibular anterior teeth. Figure 2 highlights peculiar mucosal lesions that the patient developed during her course of treatment.

The onset of the oral tongue lesions was insidious, with areas of desquamation located on the lateral aspects of the tongue. There were heavy deposits of dental plaque and calculus located primarily on the lingual aspect of the mandibular anterior teeth. Also, there was no evidence of any cutaneous manifestations. The patient was subjected to oral prophylaxis and oral hygiene measures including antibacterial rinses (Chlorhexidine 0.2% twice daily). Although the patient felt symptomatically, there was a typical waxing and waning pattern noted with respect to the mucosal lesions during the course of treatment.

## 3 Differential Diagnosis, Investigations and Treatment

During the time of presentation, her hemoglobin was 13 g/dL (06/02/23, normal); Folate: 5 ng/mL (15/02/22, normal); Vitamin B12: 180 pg/mL (15/02/22, slightly decreased); serum ferritin: 213.78 ng/mL (15/02/22, normal), indicating there were no nutritional deficiencies around the time of presentation. The oral features were indicative of Benign migratory glossitis (BMG).

The patient was on two chemotherapy drugs: Bevacizumab and erlotinib. In case of erlotinib, an epidermal growth factor receptor inhibitor (EGFR), the oral aphthous ulcerations are invariably associated with the characteristic cutaneous papulopustular mucosal changes [1]. The patient developed the oral mucosal changes around 12 months from the initiation of the angiogenesis inhibitor, Bevacizumab, which is a monoclonal antibody directed against Vascular‐endothelial growth factor (VEGF). Hubiche et al. have reported similar drug‐induced mucosal changes [3].

The patient was managed symptomatically. Her cancer treatment continued without any interruption.

## 4 Discussion

BMG is characterized by white serpiginous striae that surround areas of depapillation (red, desquamated zones). Inhibition of VEGF expression in patients with bevacizumab disrupts the reparative capability of the oral tongue epithelium that predisposes them to develop BMG [1, 3]. There are very few literary anecdotes that relate BMG to bevacizumab administration [1, 3]. In line with the existing literature, the patient developed the lesions almost after 8–12 months of administration of the targeted therapy. Also, they were associated with a burning sensation, in contrast to the painless variant that has been normally reported in nonbevacizumab associated BMG.

## 5 Conclusion

It is pertinent for the oncologists to be aware of these potential adverse mucosal changes in patients on bevacizumab and other anti‐angiogenic drugs. Special focus on oral hygiene measures is unquestionably important to palliate and minimize the discomfort.

## Author Contributions

**Sandeep Gurav:** conceptualization, project administration, supervision, writing – review and editing. **Gurkaran Preet Singh:** conceptualization, methodology, supervision, validation, visualization, writing – review and editing. **Radhika Jain:** conceptualization, data curation, formal analysis, visualization, writing – original draft.

## Funding

The authors have nothing to report.

## Consent

The Patient's son provided written informed consent for the publication of the case details.

## Conflicts of Interest

The authors declare no conflicts of interest.

## Data Availability

The data that support the findings of this study are available from the corresponding author upon reasonable request.
